# The effect of triple CFTR modulator therapy and azithromycin on ion channels and inflammation in cystic fibrosis

**DOI:** 10.1183/23120541.00502-2024

**Published:** 2024-12-16

**Authors:** Suhad Bani Melhim, Lisa E.J. Douglas, James A. Reihill, Damian G. Downey, S. Lorraine Martin

**Affiliations:** 1 School of Pharmacy, Queen's University Belfast, Belfast, UK; 2 Department of Clinical Pharmacy and Pharmacy Practice, Faculty of Pharmaceutical Sciences, The Hashemite University, Zarqa, Jordan; 3 Wellcome-Wolfson Institute of Experimental Medicine, School of Medicine, Dentistry and Biomedical Science, Queen's University Belfast, Northern Ireland, UK

## Abstract

**Background:**

Inflammation in cystic fibrosis (CF) airways is difficult to treat with well-established regimens often including azithromycin (AZ) as an immunomodulatory drug. As AZ has been reported to require CF transmembrane conductance regulator (CFTR) to be able to reduce interleukin (IL)-8 and given the emergence of highly effective CFTR “triple” modulator therapy (elexacaftor/tezacaftor/ivacaftor; ETI), the aim of this study was to investigate the effect of AZ and ETI, singly and in combination, on ion channel activity and to assess the potential anti-inflammatory effects.

**Methods:**

Electrophysiological assessment of ETI and AZ was performed on three-dimensional cultures of primary CF human bronchial epithelial (HBE) cells using a Multi Trans-Epithelial Current Clamp. IL-8 from NuLi-1 (non-CF) and CuFi-1 (CF) cells treated with AZ was measured by ELISA. Inflammatory mediators from primary CF HBE cells exposed to tumour necrosis factor-α in the presence of AZ, ETI and their combination, were screened using the Proteome Profiler™ Human Cytokine Array Kit, with selected targets validated by ELISA.

**Results:**

AZ did not alter CFTR chloride efflux, nor did it have any synergistic/antagonistic effect in combination with ETI. AZ reduced IL-8 in NuLi-1 but not CuFi-1 cells. The Proteome Profiler™ screen identified several disease-relevant cytokines that were modulated by treatment. Subsequent analysis by ELISA showed IL-8, IL-6, CXCL1 and granulocyte–macrophage colony-stimulating factor to be significantly reduced by treatment with ETI, but not by AZ.

**Conclusions:**

Incorporating ETI into the standard of CF care provides an opportunity to re-evaluate therapeutic regimens to reduce treatment burden and safely discontinue chronic treatments such as AZ, without loss of clinical benefit. Identification of redundant treatments in the era of CFTR modulation may improve medication adherence and overcome potential adverse effects associated with the chronic use AZ and other drugs.

## Shareable abstract

**Elexacafor/tezacaftor/ivacaftor reduced pro-inflammatory cytokines in primary CF HBE cells. Combination with azithromycin showed no further improvements on both inflammatory cytokine and ion channel activity. Importantly, no antagonist effect was observed.** https://bit.ly/3yVQubE

## Introduction

Airway inflammation is a hallmark of cystic fibrosis (CF) and plays a key role in the progression of lung injury. It is driven by mutations in the CF transmembrane conductance regulator (CFTR) gene, which leads to reduced chloride secretion [1]. This, coupled with an aberrant overactivity of epithelial sodium channels (ENaC), leads to hyper-absorption of water from the airway lumen. The subsequent dehydration of the airway surface liquid and impaired mucociliary clearance (MCC), predisposes people with CF (pwCF) to devastating cycles of chronic infection and inflammation [2].

Triple CFTR modulator therapy, consisting of two CFTR correctors, elexacaftor (ELX/VX-445) and tezacaftor (TEZ/VX-661) and a CFTR potentiator, ivacaftor (IVA/VX-770) (ETI) (known as Kaftrio, UK/European Union (EU) and Trikafta, USA), improves the processing, trafficking and gating of CFTR proteins, thereby increasing the amount of functional CFTR at the cell surface [3]. ETI is an approved treatment for pwCF aged 2 years and older with at least one F508del mutation and has the potential to treat 90% of the CF population [4, 5].

ENaC and CFTR have been reported to be physically associated in airway epithelial cells. The presence of CFTR in normal cells can reduce ENaC activity by regulating the channel open probability, preventing proteolytic activation of ENaC; in CF, however, mutated CFTR fails to maintain this regulation [6, 7]. Additionally, CFTR and Ca^2+^-activated chloride channels (CaCC)/TMEM16A seem to be interdependent in terms of ion current activation as well as membrane expression [8]. Chronic treatment with IVA has been shown to reduce ENaC and CaCC activity in primary CF human bronchial epithelial (HBE) cells (F508del/G551D), with a similar effect also reported for IVA in combination with lumacaftor (LUM) in normal and F508del/F508del CF HBE, suggestive of an off-target effect of CFTR modulators [9]. However, little research has been carried out on the effect of CFTR restoration by the triple therapy, ETI on other ion channels [10].

In a CF study looking at chronic infection and inflammation, IVA led to a reduction in sputum neutrophil elastase, interleukin (IL)-8, and IL-1β levels in the first week of treatment that was sustained for 2 years [11], as well as a reduction in blood IL-6 levels [12]. LUM/IVA reduced (C-X-C motif) ligand 8 (CXCL8), CXCL1 and CXCL2 transcripts as well as p38 mitogen-activated protein kinases phosphorylation in response to *Pseudomonas aeruginosa* exposure in primary differentiated CF HBE cells [13]. LUM/IVA and TEZ/IVA have also been reported to reduce IL-18, TNF-α and caspase-1 and increased the anti-inflammatory cytokine IL-10 levels in lipopolysaccharide (LPS)/ATP-stimulated peripheral blood monocytes (PBMCs) isolated from pwCF [14]. In pwCF, serum IL-18 and TNF decreased significantly after 3 months of LUM/IVA treatments, but IL-1β only decreased following TEZ/IVA [10, 14].

ETI has been found to significantly reduce the levels of six different ceramides in CFBE41o- cells, which accumulate in CF epithelial cells and are known to induce inflammation [15]. Additionally, ETI was reported to increase CFTR protein expression and reduce ATP/P2X7R-induced inflammasome activation in CF monocytes as well as IL-1β secretion [16]. The impact of ETI on CF airway epithelial cell inflammation, however, requires further investigation [17].

The immunomodulator drug azithromycin (AZ) has historically shown clinical benefit for pwCF [18]. AZ has a bacteriostatic function that involves the reduction of bacterial pathogenicity, including *P. aeruginosa.* Although the mechanisms of action are not completely understood, AZ use in pwCF not infected with *P. aeruginosa* has also been associated with a significant reduction in the number of pulmonary exacerbations and inflammatory markers [19]. These include immunomodulatory effects and anti-inflammatory properties that can aid the alleviation of some of the symptoms associated with progressive lung damage in pwCF [20]. Moreover, AZ has been reported to reduce IL-8 in tumour necrosis factor (TNF)-α-stimulated HBE cells transfected with CFTR (IB3-1/S9) but not CF cells (IB3; F508del/W1282X) suggestive of a link between the AZ-mediated reduction of IL-8 and functional CFTR [21].

ENaC inhibition continues to represent an attractive therapeutic strategy in CF even though current ENaC inhibitors and channel blockers have not yet yielded clinical success. In ENaC-overexpressing HBE cells, AZ has been shown to supress ENaC [2, 22] and has been reported to restore chloride transport upon 96 h of treatment with AZ in CF airway epithelial cells [23, 24]. These observations have not, however, been confirmed using suitable *ex vivo* models, such as well-differentiated CF HBE three-dimensional (3D) cultures grown at the air–liquid interface (ALI), nor has the effect of CFTR correction by ETI in combination with AZ been studied.

As more pwCF gain access to ETI it will be increasingly important that clinicians understand the efficacy or antagonism of multidrug combinations on the airway. Herein, we explore the hypothesis that AZ may have a synergistic influence on ENaC and CFTR activity in combination with ETI using primary CF 3D cultures of airway epithelial cells and examine the effect of AZ, alone and in combination with ETI, on airway inflammation.

## Materials and methods

### Cell culture

The cell lines, HBE NuLi-1 (ATCC® CRL-4011™) and CuFi-1(ATCC® CRL-4013™), which carries F508del/F508del CFTR genotype, were obtained from the American Tissue Culture Collection [25, 26]. Primary CF HBE cells from two donors (obtained under Institutional Review Board-approved protocols) were kindly provided by Robert Bridges, Rosalind Franklin University of Medicine and Science, (North Chicago, USA). A further two CF HBE donors were obtained from Epithelix SaRL, (Switzerland) and from the CF Translational Research Centre at McGill University (Montreal, QC, Canada). All primary CF HBE cells were of a homozygous (F508del/F508del) genotype. Cells were cultured in Airway Epithelial Cell Basal Medium (PromoCell®, Heidelberg, Germany) containing a supplement mix and 1% penicillin–streptomycin (10 000 U·mL^−1^) (Thermo Fisher, UK) on collagen-coated T75 flasks incubated at 37°C/5% CO_2_.

### Air–liquid interface culture

Briefly, primary CF HBEs were grown using PneumaCult-Ex Plus Medium with PneumaCult™-Ex Plus Supplement, hydrocortisone (STEMCELL Technologies, Vancouver, Canada) and penicillin–streptomycin 1% (v/v). Cells were seeded at a density of 1.7×10^5^ cells per filter onto HTS Transwell 24-well filter inserts (Corning Inc., Corning, NY, USA). Upon confluency, cells were transitioned to the ALI by leaving the apical chamber dry and switching the basal chamber medium to PneumaCult™-ALI Maintenance Medium (STEMCELL Technologies), which was prepared according to the manufacturer's protocol, with the addition of 2% (v/v) PALL Ultroser^TM^ G (Sartorius, Göttingen, Germany) and 1% (v/v) penicillin–streptomycin (Sigma-Aldrich, Gillingham, UK). The cells were well differentiated and ready to use in experiments 4 weeks after airlift.

### Electrophysiological assessment of ion channel activity of CF HBE cells treated with AZ and ETI

To assess ion channel activity, vehicle control (VC; 0.08% (v/v) dimethylsulfoxide (DMSO); VWR Life Sciences, Lutterworth, UK) and AZ (10 µg·mL^−1^; Sigma-Aldrich) [27] were added basolaterally to ALI inserts and incubated for 24 and 96 h. For the latter, ETI (VX-445/VX-661/VX-770 at 3/18/1 µM; Adooq Bioscience, Irvine, CA, USA) was added to relevant wells for the final 24 h only [28]. The medium was then replaced and the cells were bathed bilaterally for 1 h at 37°C and 5% CO_2_ in sterile F12 Coon's Modification powder (Sigma-Aldrich) dissolved in 1 L distilled water supplemented with 20 mM 4-(2-2-hydroxyethyl)-1-piperazine ethanesulfonic acid (HEPES; Sigma-Aldrich) and 1% (v/v) penicillin–streptomycin (10 000 U·mL^−1^), pH adjusted to 7.4 using 1 M Tris-HCl. The 24-well HTS Transwell plate was then mounted onto a Multi Trans-Epithelial Current Clamp (MTECC) system (EP Devices, Bertem, Belgium), which measures trans-epithelial resistance (RT) and open circuit potential (PD) across well-differentiated epithelial tissue, enabling calculation of equivalent short circuit current based on Ohm's law (I=V/R) where I=current, V= voltage and R=resistance. Once the baseline current was established, apical treatment with amiloride (10 µM; Sigma-Aldrich), forskolin (20 µM; Sigma-Aldrich), CFTR_inh_172 (20 µM; Adooq) and UTP (100 µM; Sigma-Aldrich) were added sequentially to measure changes in equivalent current (I_eq_) according to a well-established regimen [9]. All measurements were carried out at 37°C.

### Treatment of cuFi-1 and nuLi-1 cells with AZ and determination of IL-8

CuFi-1 and NuLi-1 cells were seeded onto a collagen-coated 12-well plate (6×10^5^ cells per well) and incubated for 24 h. The cells were then pre-incubated for 30 min with AZ (0.4 and 10 µg·mL^−1^) followed by treatment with 10 ng·mL^−1^ TNF-α (R&D systems) for a further 24 h. Cell conditioned medium (CCM) was then collected for initial determination of IL-8 (Human DuoSet ELISA; R&D Systems, Abingdon, UK).

### Proteome Profiler Human Cytokine Array

The relative secretion of 36 inflammatory markers in CCM was assessed using the Proteome Profiler™ Human Cytokine Array Kit (R&D Systems, UK catalogue no. ARY005B) according to the manufacturer's instructions. Primary human CF HBE cells were seeded onto collagen-coated 12-well plates, at a seeding density of 6×10^5^ cells per well and incubated at 37°C, 5% CO_2_ for 24 h. Submerged cells were then treated with AZ (10 µg·mL^−1^), ETI (3/18/1 μM) and an AZ/ETI combination, with TNF-α (10 ng·mL^−1^; R&D systems) included as a stimulant for a further 24 h. Manipulation of different stock concentrations ensured an equal DMSO concentration (0.08%) across treatments. CCM from the different treatments were then probed on separate nitrocellulose membranes as provided in the kit.

### Validation of Proteome Profiler Human Cytokine Array results by ELISA

To validate the results from the Proteome Profiler^TM^ primary CF HBE cells from three donors were prepared as described above. CCM was collected for quantification of inflammatory mediators by ELISA to include, IL-8, IL-6, CXCL1 and granulocyte–macrophage colony-stimulating factor (GM-CSF). All ELISAs (Human DuoSet ELISAs; R&D Systems) were performed as per the manufacturer's instructions.

### Statistical analysis

Statistical analyses were performed using GraphPad Prism v.9.1.2. Data are presented as the mean, with error bars representing sem. A Kruskal–Wallis statistical test with Dunn's multiple comparisons *post hoc* test was used to compare three or more groups; *: p≤0.05, **: p≤0.01, ***: p≤0.001.

## Results

### CF HBE ion channel responses to ETI, AZ and ETI/AZ in combination in CF HBE

**FIGURE 1:**
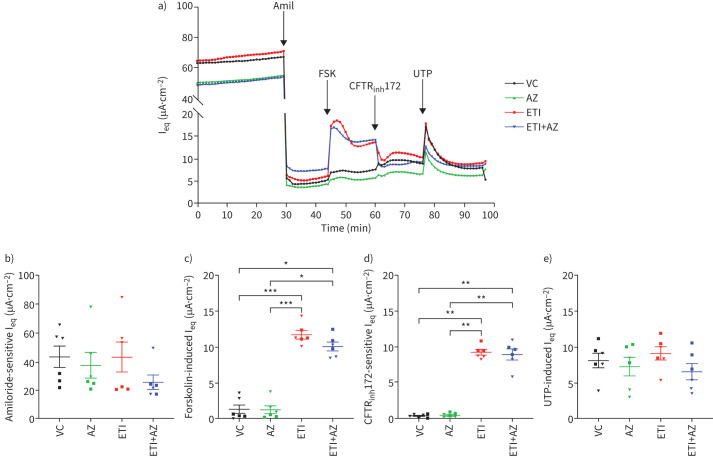
Azithromycin (AZ) has no synergistic/antagonist effect on ion channels upon combination with elexacaftor/tezacaftor/ivacaftor (ETI) in homozygous (F508del/F508del) cystic fibrosis (CF) human bronchial epithelial (HBE) cells at 24 h. a) Representative traces of equivalent current (I_eq_) over time. CF HBE primary cells grown at the air–liquid interface were pre-treated basolaterally for 24 h with vehicle control (VC) dimethylsulfoxide (0.08%; v/v), AZ (10 µg·mL^−1^), ETI (elexacaftor/tezacaftor/lumacaftor; 3/18/1 µM) and ETI+AZ in combination. Changes in I_eq_ were measured using the Multi Trans-Epithelial Current Clamp-24 system after the apical addition of amiloride (10 µM), forskolin (20 µM), CFTR_inh_172 (20 µM) and uridine-5′-triphosphate (UTP) (100 µM). Quantified b) amiloride-sensitive I_eq_; c) forskolin-induced I_eq_; d) CFTR_inh_172-sensitive I_eq_ and e) UTP-induced I_eq_ (µA·cm^−2^) are shown. Amil: amiloride; FSK: forskolin; I_eq_: . Data are presented as mean±sem. Statistical analyses were performed using a Kruskal–Wallis statistical test with Dunn's multiple comparisons *post hoc* test. *: p-value≤0.05; **: p≤0.01; ***: p≤0.001. n=6 (three filters each from two donors).

The effects of AZ and the AZ-ETI combination on ENaC, CFTR and CaCC channels were assessed by electrophysiology with sequential addition of 1) amiloride, which acts as a direct ENaC blocker; 2) forskolin, which increases cyclic AMP and stimulates chloride secretion; 3) CFTR_inh_172, which inhibits chloride ion secretion by CFTR; and 4) UTP, which stimulates CaCC, such as TMEM16a [7]. Representative traces at 24 h are shown in figure 1a. Treatment with either AZ or ETI alone showed no significant inhibition of amiloride-sensitive I_eq_, indicating that the drugs had no effect on ENaC (figure 1b). Similar traces were obtained at 96 h (supplementary figure 1a) with no difference observed between the various drugs or when used in combination (supplementary figure 1b).

The effect of treatment on chloride secretion through CFTR channels was verified by acute sequential addition of forskolin and CFTR_inh_172. AZ showed no effect on forskolin-induced and CFTR_inh_172-sensitive currents at 24 h nor at 96 h; however, ETI and ETI-AZ in combination were able to restore CFTR activity and chloride secretion as evident in figure 1c,d and supplementary figure 1c,d over the two time points**.** Both showed a significant increase in Forskolin-induced and CFTR_inh_172-sensitive currents (CFTR activity) compared with vehicle and AZ-treated cells. CaCC activity was assessed by measuring responses to UTP. All treatments had no inhibitory nor stimulatory activity on CaCC compared with VC at both time points (figure 1e and supplementary figure 1e).

There was no change in trans-epithelial electrical resistance (TEER) upon treatment with AZ, ETI and ETI-AZ in combination compared with VC at 24 and 96 h. This provided confidence of both cellular and tight junction integrity of CF HBE cells at the ALI upon treatment with the drugs (either singly or in combination) (supplementary figure 2a,b).

### Effect of azithromycin on IL-8 secretion by homozygous CF and non-CF HBE cells upon stimulation by TNF-α.

**FIGURE 2:**
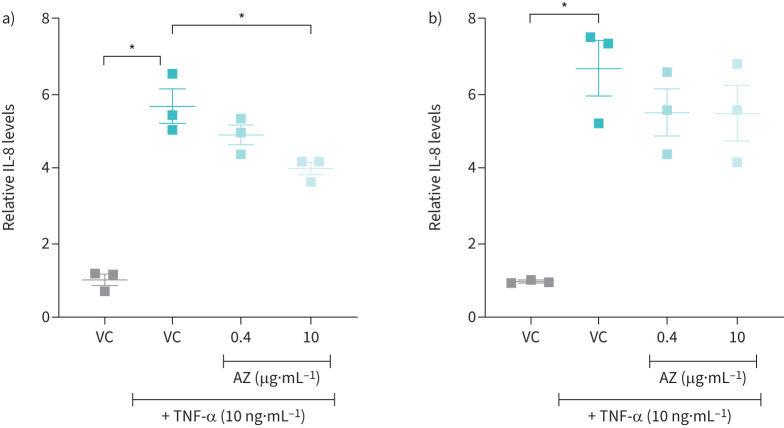
Effect of azithromycin (AZ) on tumour necrosis factor-α (TNF-α)-stimulated interleukin (IL)-8 levels in NuLi-1 and CuFi-1 cells. IL-8 levels relative to vehicle control (VC) in 24 h, cell conditioned medium collected from TNF-α (10 ng·mL^−1^)-stimulated, submerged a) NuLi and b) CuFi-1 cells (passage number 3) treated with 0.4 and 10 μg·mL^−1^ AZ for 24 h and VC (0.08% dimethylsulfoxide, v/v). n=3. Data are presented as mean±sem. Statistical analyses were performed using a Kruskal–Wallis statistical test with Dunn's multiple comparisons *post hoc* test. *: p≤0.05.

TNF-α induced a significant increase in IL-8 secretion in both NuLi-1 and CuFi-1 submerged cells. Cells were also treated with AZ (0.4 and 10 µg·mL^−1^), equivalent to peak plasma and lung peak tissue concentration, respectively [27]. A significant reduction was only apparent in NuLi-1 cells with AZ (10 µg·mL^−1^) (p≤0.05) (figure 2a,b).

### Proteome Profiler™ screen of cytokine secretion from TNF-α stimulated primary CF HBEs treated with ETI and AZ

**FIGURE 3:**
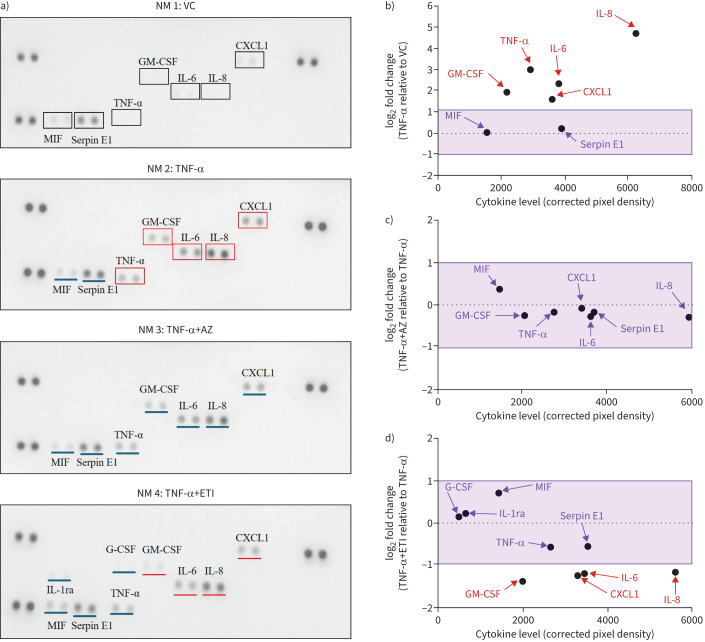
Proteome^TM^ Profiler screen of changes in cytokine production in cell conditioned medium (CCM) from homozygous (F508del/F508del) cystic fibrosis (CF) human bronchial epithelial (HBE) cells in response to tumour necrosis factor-α (TNF-α), azithromycin (AZ) and elexacaftor/tezacaftor/ivacaftor (ETI). a) Proteome Profiler^TM^ array of CCM collected from submerged primary CF HBE cells (1 donor) after 24 h of treatment. Nitrocellulose membranes (NM) 1–4 are as follows: NM1: VC (0.08% dimethylsulfoxide; v/v); NM2: TNF-α (10 ng·mL^−1^); NM3: TNF-α+AZ (10 µg·mL^−1^) and NM4: TNF-α+ETI (elexacaftor/tezacaftor/lumacaftor; 3/18/1 µM). log_2_ fold change of corrected pixel density was calculated for b) TNF-α *versus* VC c) TNF-α+AZ *versus* TNF-α and d) TNF-α+ETI *versus* TNF-α. Cytokines that exhibited log_2_-fold change ≥1 are shown in red boxes when compared to vehicle (black boxes) and underlined with a red line when compared to TNF-α. Blue line indicates log_2_ fold change between −1 and 1. MIF: macrophage migration inhibitory factor; GM-CSF: granulocyte–macrophage colony-stimulating factor; G-CSF: granulocyte colony-stimulating factor.

An unbiased view of the inflammatory cytokine landscape was conducted using a Proteome Profiler^TM^ array (figure 3a) and the log_2_ fold change of corrected pixel density to positive spots was calculated to allow for a better comparison (figure 3b–d). Cytokines that exhibited marked increases and a log_2_ fold change ≥1 upon stimulation of submerged primary human CF HBE cells with TNF-α (figure 3a (NM2)) compared with VC (figure 3a (NM1)) are marked in red boxes and include IL-8, IL-6, CXCL1, GM-CSF and TNF-α (figure 3b). When TNF-α-stimulated submerged primary human CF HBE cells were treated with AZ, little apparent change was observed in the levels of these analytes (figure 3a (NM3) and figure 3c). In contrast, a reduction was observed upon treatment of TNF-α-stimulated submerged primary human CF HBE cells with ETI, as shown by log_2_ fold changes ≤−1, with IL-8, IL-6, CXCL1 and GM-CSF (figure 3a (NM4) and figure 3d). IL-1 receptor antagonist (IL-1ra) and GM-CSF spots showed as positive on the TNF-α+ETI array membrane only. Other visible analytes included serpin E1 and MIF (macrophage migration inhibitory factor). The anti-inflammatory cytokine IL-10 was not detected on any of the array membranes.

### Validation and quantification of selected cytokines by ELISA

ELISAs to confirm results from Proteome Profiler^TM^ experiment were performed for those cytokines (*i.e.* IL-8, IL-6, CXCL1 and GM-CSF) that exhibited a log_2_-fold change ≤−1 upon treatment with ETI and were compared with samples treated with AZ and AZ in combination with ETI, all in the presence of TNF-α.

**FIGURE 4:**
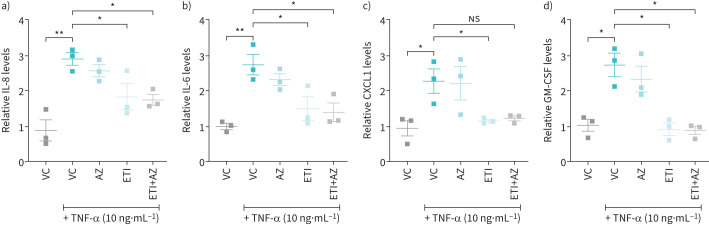
Reduction of pro-inflammatory cytokines upon treatments with elexacaftor/tezacaftor/ivacaftor (ETI) not augmented by azithromycin (AZ) in cell conditioned medium (CCM) from tumour necrosis factor-α (TNF-α)-induced homozygous (F508del/F508del) cystic fibrosis (CF) human bronchial epithelial (HBE) cells. Relative cytokine levels to untreated cells in CCM collected from submerged primary CF HBE cells after a 24-h treatment with vehicle control (VC) of dimethylsulfoxide 0.08% (v/v), AZ (10 µg·mL^−1^), ETI (ELX/TEZ/IVA; 3/18/1 µM) and ETI+AZ in combination, stimulated with TNF-α (10 ng·mL^−1^). Relative a) interleukin (IL)-8, b) IL-6, c) CXCL1 and d) granulocyte–macrophage colony-stimulating factor (GM-CSF) levels to untreated cells. Data are presented as mean ±sem. Statistical analyses were performed using a Kruskal–Wallis statistical test with Dunn's multiple comparisons *post hoc* test. n=3 from three donors. *: p≤0.05; **: p≤0.01; NS: not significant.

ELISAs using CCM collected from submerged primary human CF HBE cells (obtained from three CF donors) confirmed the ability of TNF-α to stimulate IL-8, IL-6, CXCL1 and GM-CSF, an effect that was significantly reduced by ETI (38%, 45%, 49% and 65%, respectively) (figure 4a–d). No significant reduction for the four analytes tested was observed by AZ treatment nor was any further reduction observed with AZ in the presence of CFTR correction by ETI.

## Discussion

CF results in chronic lung inflammation with increased levels of several pro-inflammatory cytokines reported in CF airways [29, 30]. As TNF-α is a crucial element in the initiation and progression of inflammation in CF involved in NF-κB activation [31, 32], it was chosen as a suitable pro-inflammatory stimulant to investigate the anti-inflammatory effects of AZ, ETI and an AZ/ETI combination. The concentration of ETI used in our *in vitro* experiments was based on Keating *et al*. [28], a study that also provided the biological and molecular rationale for the first phase 2 randomised clinical trial conducted to evaluate ETI.

We were unable to confirm an increase in chloride ion efflux by AZ. This is in contrast to reports by Oliynyk *et al.* [24], who showed an increase in chloride efflux by AZ after 96 h; albeit no effect on CFTR mRNA expression was observed. Differences include our use of MTECC, an automated electrophysiological assay system, compared with past studies that used halide-sensitive fluorescent probes (6-methoxyquinolinium derivatives) to measure Cl^−^ transport [23, 24]. Although such screening assays for CFTR are high throughput and allow rapid and cost-effective screening, they are at best semi-quantitative as the net electrochemical anion change across the membrane varies during the course of the assay [33]. Reported chloride efflux assays were also performed using submerged cultures of a CFBE cell line; in contrast, primary CF HBE cells grown in 3D cultures at the ALI were used in this study. ALI culture exhibits pseudo-stratified epithelium with a mucociliary phenotype, which can facilitate CFTR exploration, assessment of epithelial barrier functions and tissue integrity, yielding robust TEER values [34]. Importantly the model accurately reproduces the pathophysiological defect of airway dehydration in CF.

Equi *et al*. [35] assessed the potential mechanism for improved clinical outcomes of AZ in pwCF in an open-label study with subjects treated with 500 mg AZ daily for 2 weeks. Results showed that AZ treatment did not affect sodium absorption or chloride secretion. Additionally, no significant differences in CFTR mRNA, by real-time PCR and using human CF nasal epithelial (HNE) cells obtained by nasal brushings, were reported upon treatment compared with baseline levels, supportive of the results observed in this study using *ex vivo*, well-differentiated primary HBE cell ALI models. Similarly, no influence of AZ on ENaC was observed in ALI culture at 24 h in contrast to Fujikawa *et al*. [22], where an undifferentiated monolayer of ENaC-overexpressing HBE cells (β/γENaC-16HBE14o- cells with wild-type (wt)CFTR) were used and showed inhibition of ENaC.

ETI treatment of ALI cultures confirmed CFTR correction, but did not inhibit ENaC, in contrast to a report by Cholon *et al*. [9], who used IVA (5 µM) and LUM/IVA (5 µM each) over 48 h. This current study used a lower dose and shorter treatment duration of IVA (1 µM, 24 h). Additionally, the CFTR correctors, ELX and TEZ were not previously investigated. Our result also aligns with Gianotti *et al*. [36], who demonstrated that CFTR correction with LUM/IVA for 48 h did not cause a significant reduction of ENaC. Additionally, the transfection of HNE cells with wtCFTR mRNA showed no statistically significant decrease in amiloride-sensitive current at 24 and 72 h [37]. Different CFTR modulators, concentration and donors limits a robust comparison between studies, therefore, the mechanism of functional CFTR and ENaC interplay, particularly ENaC regulation by CFTR requires further investigation. We do, however, note no synergistic effect upon ETI-AZ in combination nor antagonism of CFTR correction at both the 24 and 96 h time points.

Treatment with ETI and ETI-AZ preserved CaCC activity in contrast to a previous study that reported inhibition of CaCC currents after treatment with IVA and LUM/IVA [9]. ETI maintenance of UTP-stimulated CaCC activity may constitute an advantage in CF airways [9]. AZ also had no effect on CaCC/TMEM16A similar to Oliynyk *et al*. [24], who showed that stimulated chloride efflux by AZ was not reduced upon addition of the CaCC blocker, gadolinium chloride.

AZ at concentrations equivalent to peak plasma (0.4 μg·mL^−1^) and lung peak tissue concentration (10 μg·mL^−1^) [27], were not able to significantly reduce elevated IL-8 levels observed in TNF-α-stimulated CF HBE (CuFi-1; F508del/F508del) cells (figure 2b). A significant reduction of IL-8 in wtCFTR HBE cells (NuLi-1) was, however, observed with 10 μg·mL^−1^ AZ (figure 2a). The NuLi-1 and CuFi-1 bronchial epithelial cell lines have been extensively used to generate cultures that phenotypically mimic *in vivo* airways [25]. In particular, the significant amount of Cl^−^ transport observed in NuLi-1 cells at early passages make them useful for studies of CFTR function [26]. The lack of a significant anti-inflammatory effect of AZ in CuFi-1 cells compared with NuLi-1 cells provided additional evidence to support the hypothesis that CFTR correction would enhance the anti-inflammatory effect of AZ. This was similar to a previous study which reported AZ (10 µg·mL^−1^) able to reduce IL-8 secretion in TNF-α-stimulated normal S9 cells but not in IB3-1 cells; heterozygous F508del/W1282X CFTR genotype cells [21].

The Proteome Profiler^TM^ cytokine screen provided preliminary data that treatment of submerged primary CF HBE cells with TNF-α increased several pro-inflammatory cytokines, including IL-8, IL-6, CXCL1 and GM-CSF, which could be reduced in the presence of AZ, and to a greater extent, ETI. These results, subsequently confirmed by quantification of the selected cytokines by ELISA and using cells derived from three donors, are in line with current evidence on the effect of ETI on serum and sputum inflammatory markers from pwCF. Sputum IL-8 levels were shown to be reduced in pwCF (n=79) after 3 months of ETI treatment [38]. In another CF cohort (n=48) similarly treated, a significant reduction in plasma IL-8, IL-6 and IL-17A levels and a peripheral blood immune cell composition more reflective of healthy controls was observed [39]. In those with advanced CF lung disease (n=14), 3 months of ETI treatment reduced plasma IL-6 levels alongside associated reductions in the acute phase proteins C-reactive protein and α_1_ anti-trypsin, changes that were also apparent at 1 year [40]. ETI (3 months) was also reported to significantly decrease IL-6, as well as IL-18, IL-1β and TNF-α levels in CF serum and following stimulation of isolated PBMCs by lipopolysaccharide and ATP (n=19). Corresponding mRNA levels, as well as reduced ASC (apoptosis-associated speck-like protein containing a CARD) specks and caspase-1 levels, a signature associated with downregulation of the NLRP3 inflammasome response, were also reduced in stimulated PBMCs [41]. We also now report the ability of ETI to reduce GM-CSF and CXCL1 secretion. No such reductions were observed when AZ was used alone and, no further reduction in inflammatory cytokine levels were shown in combination with ETI. Importantly, no antagonism was observed at the cellular level with the ETI-AZ combination.

One explanation for the failure of AZ to reduce inflammatory cytokines upon restoration of CFTR function could be that ETI involves post-translational modifications, which affects processing, trafficking and gating of CFTR proteins, but does not correct the CFTR gene [4]. In our own study, AZ was able to reduce IL-8 in NuLi-1 cells, an effect also observed in CFTR-transfected cells (IB3–1/S9) [21], both of which possess the wtCFTR gene. Labro *et al.* [42] reported AZ accumulation to be higher in wtCFTR tracheal epithelial cells compared with F508del mutated cells, however, the study concluded that CFTR protein is unlikely to be the macrolide carrier, proposing a transport role for a Ca^2+^/Na^+^ exchanger. Although this study was conducted in HBE cells, it should also be noted that as an immunomodulatory drug, AZ may have other potential effects on different cell types, for example, AZ accumulates in phagocytic cell lines much more than nonphagocytic cells [43], or exerts an effect on stimulated pathways other than that mediated by TNF-α, which could result in clinical benefits for pwCF [44].

A Cochrane review [18] reports oral AZ to give a 3.97% improvement in per cent predicted forced expiratory volume in 1 s (ppFEV_1_) over 6 months (n=549 patients, from four studies), compared with a >10% increase in ppFEV_1_ by ETI [4]. AZ was also recently reported to have antagonistic drug interactions with tobramycin, resulting in lower absolute improvement in ppFEV_1_ and poorer clinical outcomes compared with tobramycin alone [45]. Moreover, AZ and TEZ/IVA combination was associated with first-degree heart block after 8 months of treatment in an adult with CF, a delayed interaction that should be investigated thoroughly given TEZ/IVA is the backbone of ETI [46].

The emergence of ETI, has prompted the CF community to re-evaluate current therapeutic regimens and to assess drug combination efficacy in ETI-treated patients. The process has been initiated by the SIMPLIFY Clinical Trial Studies (NCT04378153) [47] and CF STORM trial (trial reference no. 138613), which are evaluating the impact of discontinuing hypertonic saline and dornase alfa in patients receiving ETI. A review of AZ usage (or not) in pwCF treated with ETI will further inform clinical practice and importantly, may overcome the adverse effects of chronic AZ use and its associated bacterial resistance. Potential rationalisation of regimens should be explored through clinical trials like SIMPLIFY and, if successful, could reduce drug burden and improve medication adherence.

### Conclusion

Treatment of CF primary HBEs by AZ showed no synergistic nor antagonistic effect on Na^+^ absorption by ENaC or Ca^2+^ secretion by CFTR and CaCC, either singly or in the presence of ETI. AZ also had no effect on inflammatory cytokines, even in the presence of CFTR correction by ETI. ETI treatment alone did, however, have a significant anti-inflammatory effect reducing IL-8, IL-6, CXCL1 and GM-CSF secretion in a TNF-α-induced pro-inflammatory primary CF HBE cell model, which may translate to potentially useful inflammatory markers to investigate in serum, plasma or bronchoalveolar lavage of pwCF. These studies also indicate the need for further work to elucidate the anti-inflammatory mechanism of action of ETI in CF HBE cells, as well as its effect on other cell types such as immune cells, which are instrumental in orchestrating the anti-inflammatory response in CF airways.

## Supplementary material

**Please note:** supplementary material is not edited by the Editorial Office, and is uploaded as it has been supplied by the author. Supplementary material 00502-2024.SUPPLEMENT
