# Supplementary material for: The effect of triple CFTR modulator therapy and azithromycin on ion channels and inflammation in cystic fibrosis
Source: ERJ Open Res. 2024 Dec 16;10(6):00502-2024. doi: 10.1183/23120541.00502-2024 (PMC11647873; doi:10.1183/23120541.00502-2024)
Supplement: Supplementary file 1 [file 00502-2024.SUPPLEMENT.pdf]

THE EFFECT OF TRIPLE CFTR MODULATOR THERAPY AND AZITHROMYCIN ON ION CHANNELS AND INFLAMMATION IN CYSTIC FIBROSIS

Suhad Bani Melhim[1,2], Lisa E.J. Douglas[1], James A. Reihill[1], Damian G. Downey[3], S. Lorraine Martin[1*].

[1]School of Pharmacy, Queen's University Belfast, Northern Ireland, UK.
[2]Department of Clinical Pharmacy and Pharmacy Practice, Faculty of Pharmaceutical Sciences, The Hashemite University, Zarqa, Jordan.
[3]Wellcome-Wolfson Institute of Experimental Medicine, School of Medicine, Dentistry and Biomedical Science, Queen's University Belfast, Northern Ireland, UK.

Supplementary Data

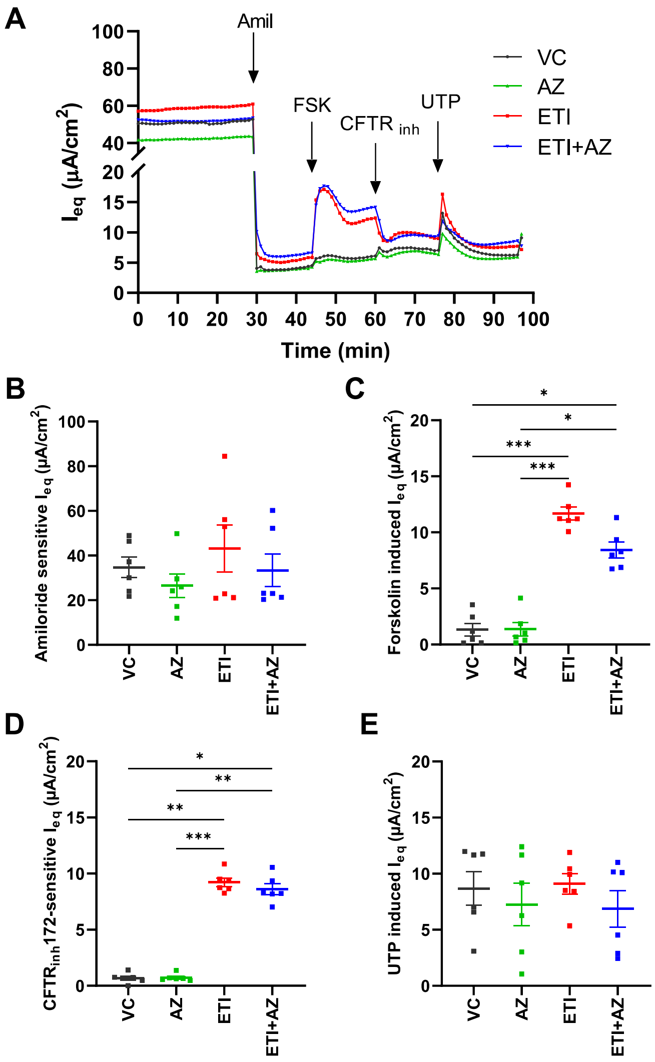

Figure S1: AZ has no synergistic/antagonist effect on ion channels upon combination with ETI in homozygous (F508del/F508del) CF HBE cells at 96 hours. (A) Representative traces of I_{eq} over time. CF HBEs primary cells grown at air liquid interface were pretreated basolaterally for **96 hours** with: vehicle (DMSO 0.08%; v/v) and AZ (10 µg/mL). ETI (ELX/TEZ/LUM; 3/18/1 µM) was added to all assigned wells 24 hours prior to experiment. Changes in I_{eq} were measured using the MTECC-24 system after the apical addition of amiloride (10 µM), Forskolin (20 µM), CFTR$_{inh}$172 (20 µM) and UTP (100µM). Quantified (B) Amiloride sensitive Ieq; (C) Forskolin induced Ieq; (D) CFTRinh172 sensitive Ieq and (E) UTP sensitive current responses (µA/cm^2) are shown. Data presented as the mean ±SEM. Statistical analyses were performed using a Kruskal-Wallis statistical test with Dunn's multiple comparisons post-hoc test. * p value * ≤ 0.05. N=6 (3 filters each from two donors).

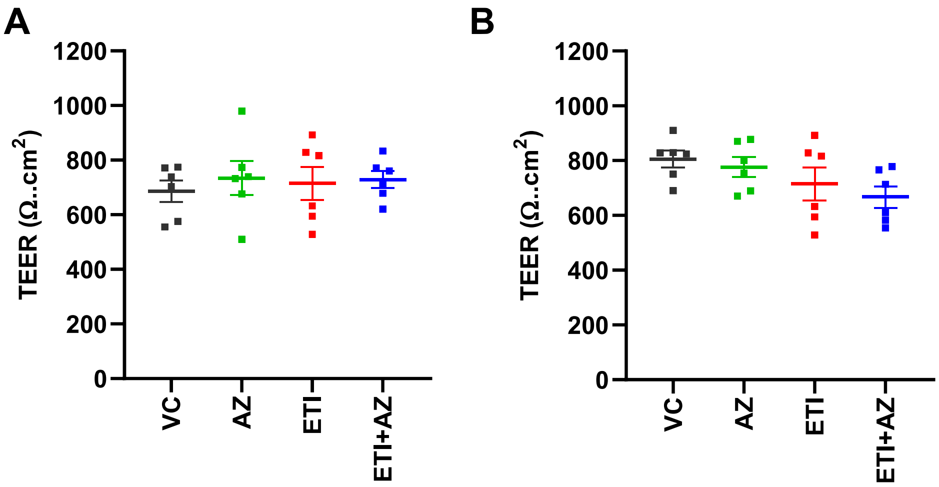

Figure S2: TEER assessment of homozygous (F508del/F508del) CF HBE cells over the course of the treatment period. CF HBEs primary cells grown at air liquid interface were pretreated basolaterally for (A) 24 and (B) 96 h with: vehicle (DMSO 0.08%; v/v) and AZ (10 µg/mL). ETI (ELX/TEZ/LUM; 3/18/1 µM) was added to all assigned wells 24 hours prior to measurement. TEER values (Ω.cm^2) were measured using the MTECC-24 system. Data presented as the mean ±SEM. Statistical analyses were performed using a Kruskal-Wallis statistical test. No significant difference was reported. N=6 (3 filters each from two donors).
